# Cumulative risk assessment of the dietary heavy metal and aluminum exposure of Finnish adults

**DOI:** 10.1007/s10661-023-11427-y

**Published:** 2023-06-07

**Authors:** Johanna Suomi, Pirkko Tuominen

**Affiliations:** Finnish Food Authority, Risk Assessment Unit, Helsinki, Finland

**Keywords:** Cadmium, Lead, Arsenic, Mercury, Nickel, Aluminum, Cumulative exposure, Risk assessment, Dietary exposure

## Abstract

While directly comparable toxicological data are unavailable, this pilot study used published toxicological endpoints for neurological damage to weigh the relative importance of cadmium, lead, arsenic, mercury, nickel, and aluminum in the mixture Finnish adults receive from their daily diet. In addition, the effects of a selection of these chemicals on cognition, kidney tubular damage, and fertility were assessed using the toxicological endpoints available in the Chemical Mixture Calculator developed by the Technical University of Denmark. Consumption data from the FinDiet 2012 national survey of 25 to 74-year-olds and occurrence data mainly obtained in national monitoring were used to estimate the cumulative dietary exposure, which was found to be so high that the possibility of neurological damage or kidney effects cannot be ruled out for most of the population, particularly fertile age women. For Finns below the age of 65 years, the main sources of cumulative exposure were bread and other cereals, non-alcoholic drinks, and vegetables. When mean exposure was statistically compared between age groups and genders, women aged 25 to 45 years had a statistically significantly higher exposure than men of the same age (*P* < 0.05) and women aged 46 to 64 years (*P* < 0.001).

## Introduction

Cumulative exposure is the combined exposure to multiple chemicals that have a common mode of action, endpoint, or target organ, and the exposure can occur from the diet alone or from multiple sources (FAO & WHO, 2020). The term exposure to chemical mixtures has also been used to refer to cumulative exposure. For a cumulative exposure assessment to take place, all the chemicals must have a similar effect on the human body. A tiered approach is recommended, and the accuracy of the estimates increases from tier 1, a common target organ, to higher tiers of, for example, a common mode of action. The toxicological basis for grouping chemicals determines whether an acute (short-term) or chronic (long-term) assessment is required (FAO & WHO, 2020). The lack of data from toxicological studies is the main challenge in the regulation of chemical mixtures (Hernández & Tsatsakis, 2017), and it also limits scientific assessments. Technically, the cumulative assessment group (CAG) of chemical substances with either a common mode of action or a common target organ can contain chemicals from many different groups, such as environmental contaminants, food additives, and pesticides, if their effect in the human body is the same.

Risk assessments of the exposure of Finnish consumers to one toxic metal/metalloid at a time have been published as part of our previous research (Suomi et al., 2020, 2021). However, heavy metals and aluminum cause damage to many of the same organs, and at least in some cases, their mode of action is suggested to be the same, occurring through the production of oxidative stress (e.g., according to many of the studies referenced by EFSA in their risk assessments of these chemicals). Therefore, these chemicals should also be assessed as a mixture, corresponding to the real-life exposure of the consumer.

Many toxic metals and metalloids have been shown to damage nerves at high enough concentrations, although the evidence for such damage varies. While the mechanism is not often known, it is possible that simultaneous exposure to several of these chemicals produces an additive or even a synergistic effect. The cumulative effect of several heavy metals was reported by de Burbure et al. (2006) to increase the occurrence of kidney damage, while Dudka et al. (2014) reported changes in blood lipoproteins and other biomarkers.

The Agency for Toxic Substances and Disease Registry (ATSDR, 2004, 2006) has published information on whether co-exposure to two metals at a time has an additive (1 + 1 = 2), synergistic (1 + 1 > 2), or antagonistic (1 + 1 < 2) effect on the damage they cause to different organs. A synergistic effect was reported for arsenic and lead, irrespective of which chemical was given first, as well as for lead and cadmium, which were only investigated with the addition of cadmium following lead exposure. An additive effect was reported for lead and methyl mercury irrespective of which was given first.

Based on this information, it is possible that simultaneous exposure to several heavy metals is more harmful than risk assessments only concentrating on one chemical at a time would suggest. On the other hand, ANSES (2021) considered that at low doses, taking into account the uncertainties inherent in the risk assessment process itself, interaction between chemicals (synergy or antagonism) is negligible. Likewise, European Food Safety Authority (EFSA) Scientific Committee et al. (2019) recommended dose addition as the default model in the absence of data proving that response addition is more appropriate for a specific case. Dose additivity assumes that all chemicals in the mixture have the same target organ and/or mode of action, and only their toxic potential differs.

The tolerable weekly intake for methyl mercury (EFSA, 2012a) and the lowest benchmark dose for lead (EFSA, 2010) have been determined on the basis of damage to the developing nervous system of fetuses and young children. The neurotoxicity of these heavy metals has been evidenced in humans by the loss of cognitive function, i.e., a decrease in the intelligence quotient. The tolerable weekly intake for aluminum (EFSA, 2008) is also based on damage to the central nervous system and the resulting loss of cognitive function, although the data are from animal tests, and corresponding dose–response data for humans are not publicly available. The central nervous system toxicity of nickel exposure has also been examined on animals (Lamtai et al., 2018), but no dose–response data are available concerning humans.

The focus of this article is on utilizing the principles of the Chemical Mixture Calculator developed by the Technical University of Denmark (Chemical Mixture Calculator (DTU), 2021) for the cumulative assessment of a group of heavy metals and aluminum. The Chemical Mixture Calculator includes databases of hazard and exposure estimates for many chemicals. In this study, we utilized the raw data of our previously published exposure estimates for six contaminants among Finnish adults (Suomi et al., 2020, 2021). The individual level estimates were used to identify which age group–gender combinations have the highest hazard index for three different endpoints. We also identified the main sources of cumulative exposure.

In addition, we assessed in a pilot study the neurotoxicity of a mixture of cadmium (Cd), lead (Pb), inorganic arsenic (iAs), methyl mercury (MeHg), nickel (Ni), and aluminum (Al), which Finnish adults are exposed to through their diet. There are currently no directly comparable toxicological data (same species, same endpoint) for all these chemicals. Therefore, toxicological endpoints for cognitive effects (Pb, MeHg, Al, Ni) as well as for peripheral nervous system damage (Cd, iAs) were used. The mechanism of action is unclear, but as exposure to these heavy metals is linked to oxidative stress via reactive oxygen species, our unproven hypothesis was that neurotoxicity occurs through oxidative stress and that it can also reach the central nervous system. Since the hypothesis could not be proven with the toxicological data available to us, this assessment included a higher level of uncertainty than assessments based on the Chemical Mixture Calculator data on hazards, and its results must be considered indicative until more information is available. In both approaches, the dose-addition model was used.

## Materials and methods

### Data and programs used for exposure assessment

This cumulative exposure assessment built on our previous work. The current study used raw data on the individual exposure of consumers to cadmium (Cd), lead (Pb), inorganic arsenic (iAs), methyl mercury (MeHg), inorganic mercury (iHg), nickel (Ni), and aluminum (Al), which were determined as part of our published work (Suomi et al., 2020, 2021).

The main part of the occurrence data used in the assessment of exposure to single chemicals comprised national monitoring data, supplemented by a smaller dataset of self-monitoring data from Finnish industry, provided by the Finnish Food and Drink Industries’ Federation, as well as literature data mainly collected from the reports of the European Food Safety Authority (EFSA, 2012a, b, c, 2014, 2015), being average occurrence values in monitoring data collected from EU Member States. In these monitoring data, the measured concentrations may be somewhat higher than the average levels in the food available on the market, as the sampling is directed to possible risky products. A food subgroup level summary of the final occurrence data and the average concentrations therein is available in Appendix 2 of the report of Suomi et al. (2020).

Most of the data on arsenic and all the data on mercury were measured as total arsenic and total mercury. Arsenic speciation results were only available for rice. For other foods, the proportion of total arsenic consisting of iAs was estimated to be 2% in fish and 3.5% in crustaceans and mollusks, 100% in water, and 70% in other foods, as described in our previous publications (Suomi et al., 2020, 2021). For methyl mercury (MeHg), the same conservative assumptions were used as in EFSA (2012a): MeHg is only found in fish and seafood; in fish, 100% of the measured total mercury is MeHg, while in crustaceans and mollusks, 80% of total mercury is MeHg. National occurrence data on aluminum as a contaminant were scarce, since its monitoring is not required in EU legislation on foodborne contaminants, and the results concerning aluminum therefore leaned more heavily on literature data than the other studied chemicals. Aluminum exposure from food additive sources was not included in the assessment since the measured occurrence data from food additive use were lacking.

In the current study, the lower bound estimate (LB) was used in the exposure assessment, which means that all analysis results below the limit of quantification (LOQ) were calculated as zeros. The lower bound estimate gives the minimum of the exposure with a given dataset, and in the case of ubiquitous environmentally occurring chemicals such as heavy metals, it is likely to underestimate the exposure. However, the analysis methods used for separate chemicals in different sample matrices differ widely in their sensitivity. Therefore, the authors considered that the known underestimate due to LB estimation would minimize the confounding effect of different limits of quantification.

The food consumption data used for the assessment of exposure to single chemicals were collected in the FinDiet 2012 survey with 48-h recall from people between the ages of 25 and 74 years and living in five areas in different parts of Finland. Details are provided in the report of Helldán et al. (2013), and the statistical indicators of the aggregated data are available in the EFSA Comprehensive Food Consumption Database (www.efsa.europa.eu/en/data-report/food-consumption-data). The dataset comprised 1295 people aged 25 to 64 years, out of whom 265 males and 356 females were between the ages 25 and 45 years, and 413 people were aged 65 to 74 years. The food consumption data were received from the Finnish Institute for Health and Welfare and were already calculated to the food ingredient level for each consumer and each survey day. Individual data at the day level were used in the calculations, and the weight as well as age and gender of the individuals were also known. The food consumption data comprised the whole diet of the studied population, and thus the references in this article to cereal products, for example, cover all types of grains such as wheat, oats, rye, barley, and buckwheat, as well as all breads and other products made from these grains. The assessments were conducted at the most detailed level of food grouping.

Lower bound exposure assessment for one chemical at a time was performed with the online program MCRA (MCRA, 2016) using the settings discussed in more detail in Suomi et al. (2021). Briefly, food types with only non-detect measurements were not included in the assessment, and for food types with some numerical and some non-detect measurements, a lower bound substitution was used where the non-detects were calculated as zeros. The beta-binomial normal exposure assessment model with logarithmic transformation was applied, and each analysis comprised 100,000 Monte Carlo simulations. For uncertainty analysis with the bootstrap method, 10,000 iterations per resampled set and 100 resample cycles (with both concentrations and individuals resampled) were used.

The exposure data of each individual for each chemical were used as the dataset for the cumulative assessment in the current study.

### Toxicological endpoints used in the DTU Chemical Mixtures Calculator

The reference values in the Chemical Mixture Calculator, developed by the Technical University of Denmark (Boberg et al., 2021; Chemical Mixture Calculator (DTU), 2021), were utilized in the assessments marked “DTU.”

The database of the Chemical Mixtures Calculator (DTU, 2021) includes the Derived Tolerable Doses (DTD) for three relevant toxicological endpoints for several of the studied chemicals. The DTD was defined (Boberg et al., 2021) as the dose level to which a person can be exposed over a lifetime without an appreciable risk of effect defined by each common assessment group, CAG. The endpoints were effects on cognition, i.e., the neurotoxic endpoint, tubular cell degeneration, i.e., the nephrotoxic (kidney toxicity) endpoint, and effects on fertility or the reproductive organs of males or females. These are referred to in the following text and tables as “neurotoxicity (DTU),” “tubular effects,” and “fertility effects.” In figures, the shorter versions “neuro (DTU),” “kidney,” and “fertility” are used. For the pilot neurotoxicity study on six chemicals described in the next section, the notations “neurotoxicity (6 chemicals)” or “neuro (6)” are used.

For a comparison of the relative weights of the different chemicals in the total cumulative exposure and its sources, we also used the DTDs as a basis for calculating the relative potency factors (RPF) as detailed in the next section. These are presented in Table 1.

**Table 1:** Derived tolerable doses and corresponding relative potency factors (RPF). The DTD values are from Chemical Mixture Calculator (DTU) (2021), and the RPF values were derived from them in the current study

Chemical	DTD (µg/kg bw/d)	RPF
Effects on cognition (neurotoxic endpoint)		
Al	100	0.0005
Pb	0.05	1.0
MeHg	0.19	0.26
Tubular cell degeneration (kidney toxicity)		
Cd	0.36	0.17
Pb	0.06	1.0
iHg	0.6	0.1
Ni	330	0.00018
Effects on fertility and/or reproductive organs		
Al	270	0.0015
Pb	0.4	1.0
iHg	1.2	0.33
MeHg	5.0	0.08

### Determining the relative potency factors for neurotoxicity (6 chemicals)

Relative potency factors (RPF) represent the toxicities of individual substances or congeners in a chemical group relative to an index compound (FAO & WHO, 2020).

The health-based guidance values were determined from either benchmark doses for a fixed increase from the baseline level, shown as a subscript of the BMDL notation (e.g., BMDL_01_), or no observed adverse effect levels (NOAELs). The NOAEL was assumed to equal BMDL_10_. As the toxicological data were not directly comparable, it was not considered necessary to adjust all the BMDL values to BMDL_10_ as the difference was assumed to be minimal after conversion to the health-based guidance value (HBGV) for human.

For cadmium, the NOAEL for peripheral neuropathy determined for mice (ATSDR, 2012) was used with a correction factor of 100 for toxicokinetic and toxicodynamic differences between rodents and humans.

For lead, the BMDL_01_ for developmental neurotoxic effects, determined as a one point (1%) decrease in the intelligence quotient scale, was determined by EFSA (2010) to be 12 µg in a liter of blood, and the corresponding dietary exposure was 0.50 µg/kg bw/d. EFSA considered a margin of exposure of 10 or more to be of negligible risk, corresponding to a daily exposure lower than 1/10 of the BMDL_01_.

The main source of methyl mercury exposure is fish, and EFSA (2012a) also considered the beneficial effects of fish fatty acids when determining the tolerable weekly intake value for MeHg. A decrease in the cognitive abilities of offspring following maternal exposure has been observed in several studies. U. S. Environmental Protection Agency (2001) refers to a BMDL_05_ range of 46–79 µg/L in maternal blood, corresponding to daily intakes of 0.857 to 1.472 µg/kg bw/d, for different neuropsychological effects on human children at 7 years of age. EFSA (2012a) reports the same BMDL_05_ of 11.5 mg Hg per kilogram of the mother’s hair. The HBGV used in the current study was the tolerable daily intake value determined by EFSA, that is, the tolerable weekly intake (TWI) divided by 7.

For humans, the NOAEL of arsenic for peripheral neuropathy has been determined as 3 µg/kg bw/d (ATSDR, 2016). This was measured as an increased loss of feeling in the fingertips and toes after exposure via drinking water. In water, arsenic is only considered to occur as inorganic salts, while food also contains organic arsenic compounds, most of which are significantly less toxic than inorganic arsenic (EFSA, 2009). The current study only considered the effect from the dietary intake of inorganic arsenic.

Lamtai et al. (2018) published a study on the behavior of rats in a Morris water maze, measuring the cognitive abilities of the animals, after dosing with nickel. Nickel exposure (intraperitoneally, 8 weeks) impaired spatial learning performance, especially in male rats. The LOAEL in the experiment was 1 mg/kg, and the NOAEL was 0.5 mg/kg. Examination of the animals also revealed increased lipid peroxidation and nitric oxide levels as well as a significant decrease in catalase and superoxide dismutase activities in the brains of both male and female rats. The authors hypothesized that increased nitric oxide formation could be one of the main reasons for nickel’s toxic effects. The corresponding dose for humans was calculated by us using a factor of 100 to correct for toxicokinetic and toxicodynamic differences between humans and rats and a factor of 3 to correct for the short duration of the experiment. In addition, only 1 to 40% of nickel received orally is absorbed, and a factor of 5% was used to convert the intraperitoneal dose to an oral dose absorbed from food. The corresponding dose was calculated for the LOAEL (for which an additional factor of 3 was used) and the NOAEL, and the average of these doses, 27.8 µg/kg bw/d, was used in the current study.

In the mouse study reported by EFSA (2008) and used for determining the tolerable intake of aluminum, the NOAEL for neurodevelopmental effects in offspring following exposure during pregnancy was 10 mg/kg bw/d. A factor of 100 was used to correct for the toxicokinetic and toxicodynamic differences between mice and humans.

The relative potency factors (RPF) presented in Table 2 were calculated from the above values by setting the RPF of the chemical with the lowest effective dose, i.e., lead, as 1 and comparing the HBGV values of all the other chemicals to that of lead.

**Table 2:** Relative potency factors (RPF) used in this study for assessing the neurotoxicity of six chemicals. The effects for which the reference doses were determined are marked A for developmental neurotoxicity/cognitive effects, and B for neuropathy. The NOAEL is the no observed adverse effect level, and BMDL is the lower confidence limit of the benchmark dose. The adjustment factor to produce the health-based guidance value (HBGV) is given in the column “Margin of exposure for low risk.” The HBGV equals the tolerable daily intake (TDI)

Chemical	Species, effect	Reference dose (µg/ kg bw/d), source	Margin of exposure for low risk	HBGV (µg/kg bw/d)	RPF
Cd	Mouse, B	200 µg, NOAEL^a^	100	2	0.025
Pb	Human, A	0.5 µg, BMDL_01_^b^	10	0.05	1
iAs	Human, B	3 µg, NOAEL^c^	1	3	0.017
MeHg	Human, A	1.2 µg, BMDL_05_^d^		0.19^e^	0.27
Ni	Rat, A	500 µg, NOAEL 1000 µg, LOAEL^f^	300 for NOAEL, 900 for LOAEL	27.8^ g^	0.0018
Al	Mouse, A	10,000 µg, NOAEL^h^	100	100	0.0005

^a^ATSDR, 2012

^b^Corresponds to 12 µg/L blood (EFSA, 2010)

^c^ATSDR, 2016

^d^Average of maternal exposure BMDL_05_ value range 0.857–1.472 µg/kg bw/d causing neurophysiological effects in children at 7 years (U.S. EPA, 2001); EFSA (2012a) also used a similar dietary exposure value to correspond to 11.5 mg/kg in maternal hair

^e^EFSA (2012a), with TWI divided by 7 for the daily dose

^f^(Lamtai et al., 2018) Intraperitoneally, with assumed 100% bioavailability. The reference dose for humans includes a factor to take into account an assumed 5% bioavailability with oral exposure

^g^Average of values calculated from the NOAEL and LOAEL

^h^EFSA, 2008

### Assessment of cumulative exposure

The individual level dietary exposure for single chemicals was assessed in our previous research, as detailed in the previous section, and in the current study, the estimates of the different chemicals were combined according to the pseudonymized ID codes of the consumers.

Cumulative exposure was calculated as the sum of exposures to individual chemicals, adjusted with their relative potency factors:

1 $$\mathrm{Cumulative\;exposure}= \sum_{i=1}^{n}{\mathrm{RPF}}_{i}\times {\mathrm{Exposure}}_{i}$$

Equation 1 was used to calculate both the individual-level dietary exposure and the mean exposure from a food subgroup. The latter was used in assessing the sources of cumulative exposure.

The hazard index was calculated using the DTD values of the Chemical Mixture Calculator (Boberg et al., 2021), Table 1, and for the pilot assessment including six chemicals, using the health-based guidance values in Table 2:

2 $$\mathrm{Hazard\;index}= \sum_{i=1}^{n}\frac{{\mathrm{Exposure}}_{i}}{{\mathrm{HBGV}}_{i}} or \sum_{i=1}^{n}\frac{{\mathrm{Exposure}}_{i}}{{\mathrm{DTD}}_{i}}$$

Hazard index values below 1 indicate a negligible risk of the health effect in question.

## Results and discussion

### Sources of cumulative exposure

**Fig. 1:**
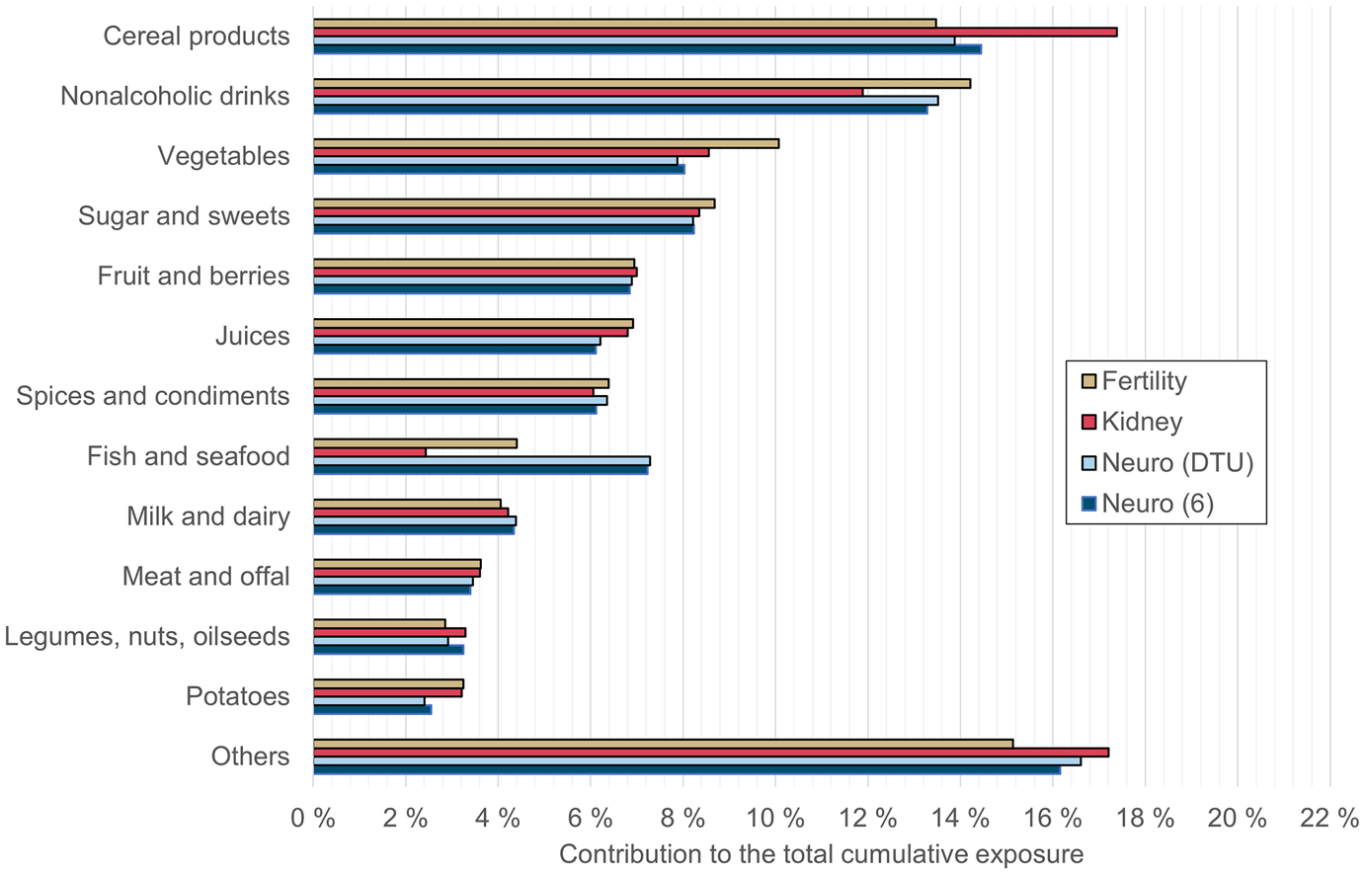
Sources of cumulative exposure for the age group 25 to 64 years. The pilot assessment of six chemicals is marked “Neuro (6),” and the other results were calculated with relative potency factors from Chemical Mixture Calculator DTD values. The group “others” includes eggs, fat, alcoholic drinks, drinking water, weight loss foods, combination foods, and food supplements. Weight loss foods contribute ca. 38% to “others”

**Fig. 2:**
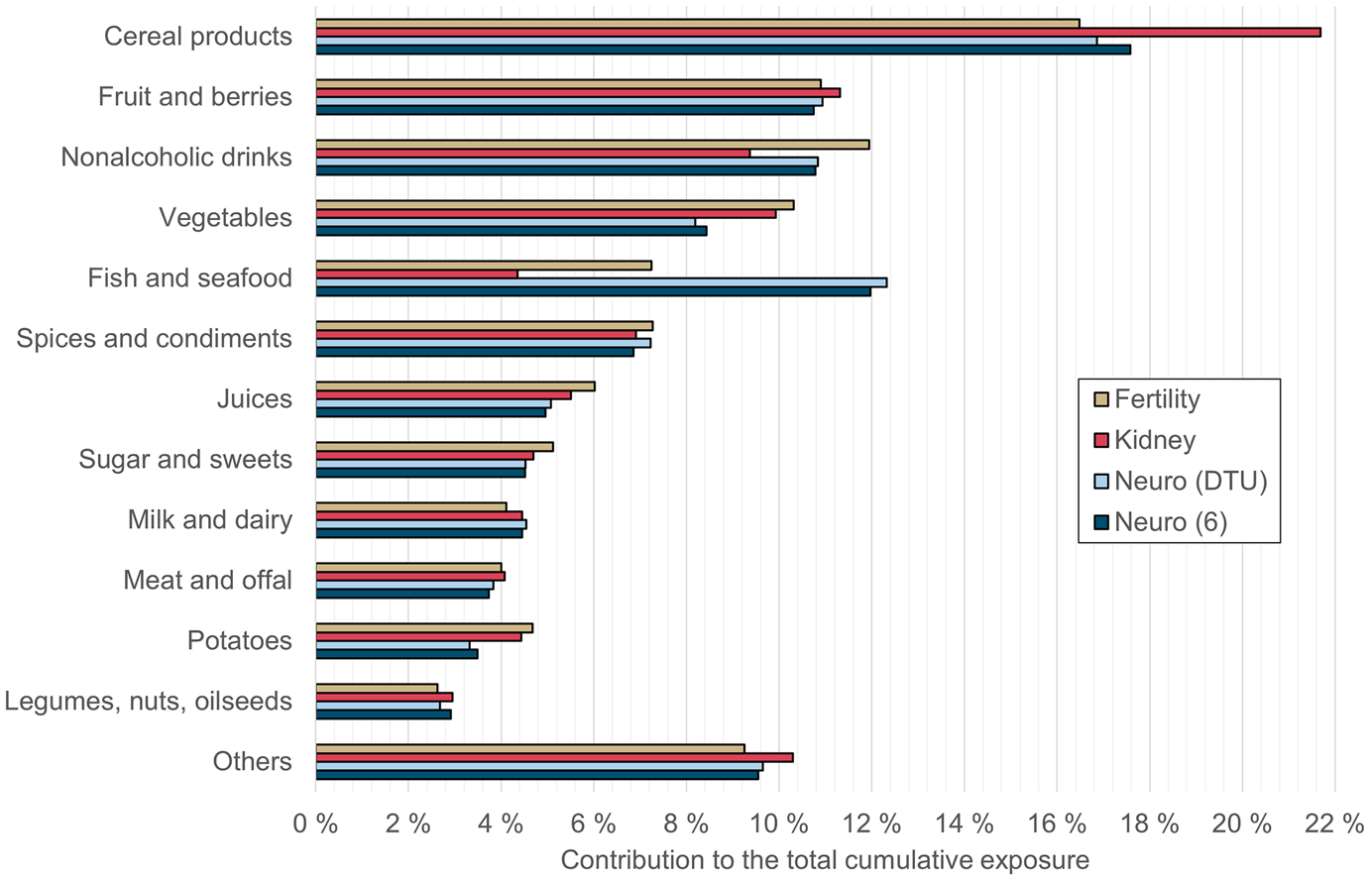
Sources of cumulative exposure for the age group 65 to 74 years. See the previous figure for an explanation of the captions. The relative contribution of weight loss foods to “others” is ca. 7%

The sources of cumulative exposure were determined for an average consumer in the age group. Sources of exposure for Finnish adults aged 25 to 64 years are presented in Fig. 1 and for elderly Finns aged 65 to 74 years in Fig. 2. Figures 1 and 2 compare the results for different endpoints included in the Chemical Mixture Calculator as well as the results of our pilot neurotoxicity study with six chemicals.

The sources of cumulative exposure in the whole diet were calculated from data on the mean exposure to each chemical from each studied food (at the level used in the raw data of the national risk assessment (Suomi et al., 2020), i.e., mainly corresponding to EFSA’s FoodEx2 grouping level 3 or higher). The sum exposure was calculated using Eq. 1, and the results were aggregated to the food group level for visual presentation.

The main food group driving cumulative exposure in both age groups and for all but one of the studied endpoints was cereal products, which includes all grains such as wheat, rye, oats, barley, and naturally gluten-free grains, as well as breads and other products made from these grains. The heavy metal and aluminum concentrations in grains and bread were not high, but their frequent and high consumption makes them an important source of exposure. For fertility effects in the younger age group, nonalcoholic drinks, including coffee, tea, soft drinks, and plant-based dairy alternatives, were estimated to be a slightly larger source than cereal products.

### Hazard index for the different endpoints

The hazard index (HI) was calculated according to Eq. 2 for neurotoxicity, kidney toxicity, and fertility effects. Table 3 details the results for 10-year age groups with the genders separated, and Table 4 presents the proportion of each age group with a hazard index above 1 for the different health effects. If HI exceeds 1, the health risk is no longer negligible.

**Table 3:** Average hazard index values for neurotoxic effects (NEURO), kidney toxicity (KIDNEY), and fertility effects (FERTILITY). The first neurotoxicity column, for the pilot study comprising six chemicals, is assessed with RPFs from Table 2, and the other three columns with DTD values from Table 1

Gender	Age group (years)	NEURO (6)	NEURO (DTU)	KIDNEY	FERTILITY
Female	25–34	4.19	3.94	3.38	0.60
	35–44	3.80	3.59	3.09	0.54
	45–54	3.33	3.11	2.65	0.47
	55–64	3.05	2.85	2.38	0.42
	65–74	2.65	2.46	2.09	0.37
Male	25–34	3.91	3.68	3.17	0.55
	35–44	3.33	3.13	2.71	0.47
	45–54	2.99	2.79	2.33	0.41
	55–64	2.83	2.63	2.22	0.39
	65–74	2.63	2.46	2.03	0.36

**Table 4:** The proportion of the population group with an (individual average) hazard index above 1, which means that a health risk cannot be ruled out. For abbreviations, please see the previous table

Gender	Age group (years)	% of the population group with HI > 1			
		NEURO (6)	NEURO (DTU)	KIDNEY	FERTILITY
Female	25–34	99%	98%	97%	10%
	35–44	97%	95%	94%	6%
	45–54	99%	98%	98%	4%
	55–64	97%	94%	92%	3%
	65–74	96%	93%	90%	1%
Male	25–34	98%	98%	97%	7%
	35–44	99%	99%	97%	4%
	45–54	97%	96%	94%	2%
	55–64	99%	94%	93%	2%
	65–74	98%	96%	92%	0%

For the average consumer, the HI values for neurotoxicity and kidney toxicity exceed the negligible risk level of 1 in all age groups. The average HI is highest for women in the youngest 10-year age group (25 to 34 years), which means that the risk of harmful health effects is highest for this group. The two youngest 10-year age groups are also those for whom the neurotoxic effect is the most dangerous, as neurological damage mostly occurs in the developing fetus. The HI was estimated for all age groups, although the effects mainly harming the development of the fetus are obviously not relevant for the elderly; they are included in the tables for comparison of the differences in exposure between age groups.

According to both our estimate with six chemicals and the estimate based on the Chemical Mixture Calculator (Chemical Mixture Calculator (DTU), 2021), more than 95% of women below the age of 45 are exposed through their diet to a heavy metal and aluminum mixture to an extent that their hazard index for neurotoxicity is raised above 1. Thus, the risk of neurological damage to the developing fetus cannot be ruled out for most of the population of childbearing age. This finding concurs with our previous assessment (Suomi et al., 2019), in which exposure to lead alone was estimated to cause an annual disease burden of nearly 600 DALY in Finland by affecting the intelligence of children.

The HI for fertility effects is below 1 for the average user in all age groups, and the possibility of heavy metals and aluminum causing damage to reproductive systems is thus low for most of the population. Only 6 to 10% of the female population of fertile age, and a lesser proportion of the male population, has a HI for fertility effects higher than one. Therefore, the subpopulation at risk of damage to the reproductive system from heavy metal and aluminum exposure is at most 10% of the adult population.

The hazard index values for neurological effects and tubular effects were higher than one in all age groups of Finnish adults. This signifies that the risk of health damage cannot be ruled out for Finnish adults or for fetuses, if the results for 25- to 45-year-old women in this study are not much higher than the typical dietary exposure of currently pregnant women or women planning to become pregnant.

The consumption data available to us did not contain a sufficient number of currently pregnant people for separate analysis, and it is therefore unknown whether the diet during pregnancy is significantly different from the diet of others in the same age group. Finnish maternity clinics offer free of charge advice on risky food, and they reach 99.7% of pregnant women as well as 99.5% of children below school age (according to Expert Statement EDK-2018-AK-189867 in the Parliament of Finland’s official documentation available online). Therefore, this advice, which includes avoiding foods high in heavy metals, is widely known, and as a result, it is likely that during pregnancy and breastfeeding, the short-term heavy metal intake of women is lower than at other times in their adult life. On the other hand, many of the heavy metals have a long half-life in the body (cadmium, lead) or can re-enter the blood from bones during pregnancy (lead), and the diet before pregnancy may therefore still influence the health of the fetus.

In interpreting the results, it is important to remember that the use of the lower bound scenario gives a minimum estimate of the cumulative effect. The accuracy of this estimate would be increased with an assessment of the dietary exposure in which the non-quantified occurrence data are not calculated as a fixed percent of the limit of quantification but as values belonging to the same distribution as the numerical values and being between 0 and the LOQ. During the assessment of the results for single chemicals, this was not available to us. Since then, our group has published a new exposure assessment model called BIKE (Ranta et al., 2021), which works under the required principles and which will be utilized in later assessments. The model will be also available as an online tool later in 2023.

### Contribution of the components to cumulative exposure

The relative contribution of the studied chemicals to the total cumulative exposure was assessed to identify, which components of the mixture had the largest effects. The age groups 25 to 64 years and 65 to 74 years had only minimal differences in the relative contributions, and Table 5 therefore presents the results in numerical form for the whole population (25 to 74 years).

**Table 5:** Relative contribution of the chemicals to the cumulative exposure (age group 25 to 74 years). A dash (-) marks the chemicals not included in the assessment of the effect in question. The row “Neurotoxicity (6)” is calculated with the RPFs in Table 2, and the other results with the DTDs in Table 1

Health effect	Cd	Pb	iAs	iHg	MeHg	Ni	Al
Neurotoxicity (6)	2%	79%	2%	–	4%	3%	9%
Neurotoxicity (DTU)	–	85%	–	–	5%	–	10%
Tubular effects	16%	81%	–	2%	–	0.3%	–
Fertility effects	–	69%	–	6%	1%	–	24%

The main contributor to all health effects was lead, which also had the highest potency (see Tables 1 and 2) according to the available toxicological data. Aluminum exposure contributed roughly one-tenth to the total neurotoxicity of the mixture and roughly one-fourth to the effects on fertility. Inorganic arsenic had a very small effect, at least with the currently available dose–response knowledge concerning its effects on the nervous system. Cadmium exposure mainly affected the kidneys, but even there, most damage was estimated to occur from lead exposure.

Previously, we have estimated that the annual burden of disease caused by the neurotoxic effects of dietary lead is 570 disability-adjusted life years (DALY) in Finland (Suomi et al., 2019). The total burden of disease caused by the neurotoxic effects of a heavy metal and aluminum mixture is higher than that of only dietary lead. In the light of the results in Table 5, where lead contributes 79–85% of the total effect of the mixture, the total burden of disease from neurotoxic effects is likely to be between 600 and 1000 DALY/year if the increase is linear, although a clearer estimate cannot be given at this stage.

### Statistical comparison between population groups

The mean cumulative exposure and the mean hazard index were compared between men and women aged 25 to 45 years, between women aged 25 to 45 years and 46 to 64 years, and between the lowest and highest weight quartile. The exposure of men and women weighing the same was also compared.

The statistical comparisons were performed using two-sided *t*-tests, in which the variances in the compared groups were assumed to be different. Tables 6, 7, and 8 present the results.

**Table 6:** Comparison of women and men aged 25 to 45 years. Two-sided* t*-test, variances assumed to be different. Each study day is used as one data point

Factor	25–45 years women	25–45 years men	*P* (25–45 years women vs. men)
Mean weight (kg)	68.9	84.5	< 0.001
Neurotoxicity, 6 chemicals			
Mean cumulative exposure (µg/kg bw/d)	0.197	0.177	0.029
Mean hazard index	3.94	3.53	
Neurotoxicity, DTU			
Mean cumulative exposure (µg/kg bw/d)	0.185	0.166	0.033
Mean hazard index	3.71	3.32	
Tubular effects			
Mean cumulative exposure (µg/kg bw/d)	0.192	0.172	0.030
Mean hazard index	3.20	2.87	
Fertility effects			
Mean cumulative exposure (µg/kg bw/d)	0.224	0.200	0.012
Mean hazard index	0.56	0.49

**Table 7:** Comparison of women aged 25 to 45 years with women aged 46 to 64 years. Two-sided *t*-test, variances assumed to be different. Each study day is used as one data point

Factor	25–45 years women	46–64 years women	P (25–45 years vs. 46–64 years)
Mean weight (kg)	68.9	72.2	< 0.001
Neurotoxicity, 6 chemicals			
Mean cumulative exposure (µg/kg bw/d)	0.197	0.159	< 0.001
Mean hazard index	3.94	3.18	
Neurotoxicity, DTU			
Mean cumulative exposure (µg/kg bw/d)	0.185	0.148	< 0.001
Mean hazard index	3.71	2.97	
Tubular effects			
Mean cumulative exposure (µg/kg bw/d)	0.192	0.149	< 0.001
Mean hazard index	3.20	2.49	
Fertility effects			
Mean cumulative exposure (µg/kg bw/d)	0.224	0.178	< 0.001
Mean hazard index	0.56	0.44

**Table 8:** Comparison of the lowest (Q1) and highest (Q4) weight quartile. In Q1, the body weights were up to 65.9 kg, and in Q4, they were higher than 87.2 kg. Two-sided *t*-test, variances assumed to be different. Each study day is used as one data point

Factor	Q1	Q4	*P* (Q1 vs. Q4)
% women in group	89.3	26.7	
Mean weight (kg)	59.2	98.9	
Mean age (years)	48.4	52.8	< 0.001
Neurotoxicity, 6 chemicals			
Mean cumulative exposure (µg/kg bw/d)	0.193	0.127	< 0.001
Mean hazard index	3.86	2.54	
Neurotoxicity, DTU			
Mean cumulative exposure (µg/kg bw/d)	0.180	0.119	< 0.001
Mean hazard index	3.60	2.38	
Tubular effects			
Mean cumulative exposure (µg/kg bw/d)	0.185	0.121	< 0.001
Mean hazard index	3.08	2.02	
Fertility effects			
Mean cumulative exposure (µg/kg bw/d)	0.218	0.141	< 0.001
Mean hazard index	0.55	0.35	

Younger men (25 to 45 years) were statistically highly significantly (*P* < 0.001) heavier than women of the same age, which is one factor explaining the statistically significantly (*P* < 0.05) higher cumulative exposure and hazard index of the women aged 25 to 45 years (Table 6). Comparing women aged 25 to 45 years with those aged 46 to 64 years, both the weight and the mean exposure, as well as the hazard index, displayed a statistically highly significant (*P* < 0.001) difference. The younger women were less heavy, and their exposure and hazard index values were higher (Table 7).

The same effect was seen when comparing mixed-gender weight quartiles (Table 8). The lowest weight quartile had a statistically highly significantly (*P* < 0.001) higher cumulative exposure than the highest weight quartile.

Men and women weighing between 69 and 71 kg were also compared. In this small group (112 data points for women and 48 for men), the distribution of weights (*P* = 0.23) and ages (*P* = 0.34) was similar, minimizing the effect of the body weight difference. The average exposure and hazard index values did not significantly differ between the two groups (*P-*value between 0.14 and 0.26).

Previously, the FinDiet 2012 report (Helldán et al., 2013) demonstrated that there is a difference between genders in the average consumption of, for example, fresh vegetables (men 84 g/d, women 94 g/d) and fresh fruit (men 100 g/d, women 142 g/d). In addition, the consumption of cereal products was reported to be higher among Finnish men aged 25 to 64 years than with women of the same age. However, in the consumption of fish foods, the difference between genders was not very large (men 50 g/d, women 40 g/d). As men are on the average heavier than women, for an assessment of whether consumption differs statistically significantly between genders, the comparison should be made between amounts consumed per kg of body weight. However, as the report focused on nutritional aspects, this comparison was not published as part of it, and it is not therefore possible to ascertain with confidence how greatly consumption differences rather than weight differences play a role in the results of the current study.

Although the subset of men and women of similar weight (69 to 71 kg) was small, the finding that their heavy metal exposure did not significantly differ indicates that weight differences can have a substantial effect on the differences in exposure. However, men and women differ statistically significantly, at least when measured as g/d, in their consumption of some food groups that we assessed to be important sources of cumulative exposure to the studied chemicals. Therefore, the difference in the assessed cumulative exposure cannot be explained by the body weights alone.

## Conclusions

The inclusion of additional chemicals (inorganic arsenic, cadmium, nickel) in the assessment of neurotoxic effects had only a small effect on the results, according to the toxicity data we were able to find on the heavy metals. The relative contribution of these three chemicals was less than 10% of the total cumulative effect of a six-chemical mixture (see Table 5).

On the other hand, the relative potency factors calculated in this study contain a large amount of uncertainty, since the health-based guidance values used to calculate them have been determined in different types of experiments and on different species (human, rat). When searching for toxicological studies, those on neurotoxicity to the central nervous system, measured as a decrease in cognitive abilities, were preferred. However, for cadmium and arsenic, we could only find information on neurotoxicity to the peripheral nervous system, detected as neuropathy. For these reasons, the results of the cumulative assessment for six chemicals are to be considered indicative until more comparable toxicological data are available.

With the Chemical Mixture Calculator approach, the cumulative dietary risk to Finnish adults from a heavy metal and aluminum mixture was found to be higher than negligible for effects on both the nervous system and the kidneys. The aluminum exposure of consumers only considered aluminum as a contaminant, since measured data on aluminum occurrence due to food additive use were not available. Thus, the total exposure to aluminum is likely to be slightly higher than that reported here, and further assessment of the exposure from all sources, including non-dietary exposure, would be needed in the future.

Based on the results presented in this study, the neurotoxicity of the heavy metal and aluminum mixture Finnish adults are exposed to in their daily diet is mainly (79–85%) driven by lead exposure. Assuming that the increase in the burden of disease from neurotoxic effects is linear, dietary exposure to the mixture is likely to cause a burden of disease of less than 1000 DALY.

In the current study, only the health risk was considered, and the results may therefore provide a pessimistic view of consumer health, as many foods that increase the heavy metal and aluminum exposure also contain chemicals that are beneficial to health. For example, even though fish is the main source of methyl mercury in the diet, it also contains fatty acids needed for neurodevelopment, and several assessments indicate that the overall effect of fish consumption is beneficial to health. In the future, there is a need for further studies with risk–benefit assessment and involving the cumulative assessment of both harmful and beneficial foodborne chemicals.

## Data Availability

The occurrence data used for exposure assessment are available as food subgroup level summaries in Appendix 2 of Suomi et al. (2020). The individual food consumption data are available according to the data management policies and procedures of the Finnish Institute of Health and Welfare THL, and aggregated consumption data are available from the EFSA Comprehensive Food Consumption Database.
